# Temporary Internal Bracing for Pediatric Odontoid Synchondrosis Fracture

**DOI:** 10.7759/cureus.17639

**Published:** 2021-09-01

**Authors:** Ayman W Taher, Cody J Falls, Paul S Page, James A Stadler

**Affiliations:** 1 Neurological Surgery, University of Wisconsin School of Medicine and Public Health, Madison, USA; 2 Orthopaedic Surgery, University of Wisconsin School of Medicine and Public Health, Madison, USA; 3 Neurosurgery, University of Wisconsin School of Medicine and Public Health, Madison, USA

**Keywords:** pediatric, internal bracing, synchondrosis, range of motion, cervical, dens, atlantoaxial, odontoid fracture, c1, c2

## Abstract

Children are predisposed to injuries of the upper cervical spine given their relatively immature osteology, ligamentous laxity, underdeveloped musculature, and larger ratios of head to body mass. Odontoid process fractures involving the synchondroses are among the most common of these injuries. Though many of these fractures can be treated conservatively with external bracing, fractures with significant displacement that are unable to be reduced require operative management. In these cases, most patients undergo C1-2 posterior fusion with arthrodesis with permanent limitation to atlantoaxial range of motion (ROM). Here, we present a novel operative approach to manage odontoid synchondrosis fractures with temporary internal bracing via C1-2 posterior instrumentation without arthrodesis. We saw a three-year-old female who presented after a motor vehicle collision with a displaced odontoid synchondrosis fracture that was unable to be adequately reduced in a closed fashion. In an attempt to preserve maximal atlantoaxial ROM, temporary internal bracing was carried out with excellent results.

## Introduction

Children are predisposed to craniocervical and upper cervical spinal injuries as a result of their large head-to-body ratio, incomplete ossification, ligamentous laxity, and underdeveloped supporting musculature [1-3]. The dentocentral and neurocentral synchondroses associated with the odontoid process are particularly susceptible to fractures in young children [4-6]. Nevertheless, odontoid synchondrosis fractures are uncommon, and optimal management of these fractures remains unclear [1,6,7].

Most children with odontoid synchondrosis fractures are managed with closed reduction and external bracing. Surgery, typically with C1-2 posterior spinal fusion, is reserved for children with significantly displaced or irreducible fractures, spinal cord injury, or failure of conservative management [8,9]. This results in permanent changes in cervical biomechanics and has been shown to decrease terminal axial range of motion (ROM) [9].

Here, we report successful management of a displaced odontoid synchondrosis fracture via temporary C1-2 instrumentation. This is a novel management option for pediatric fractures of the odontoid process with an opportunity to preserve cervical biomechanics and range of motion.

## Case presentation

**Figure 1:**
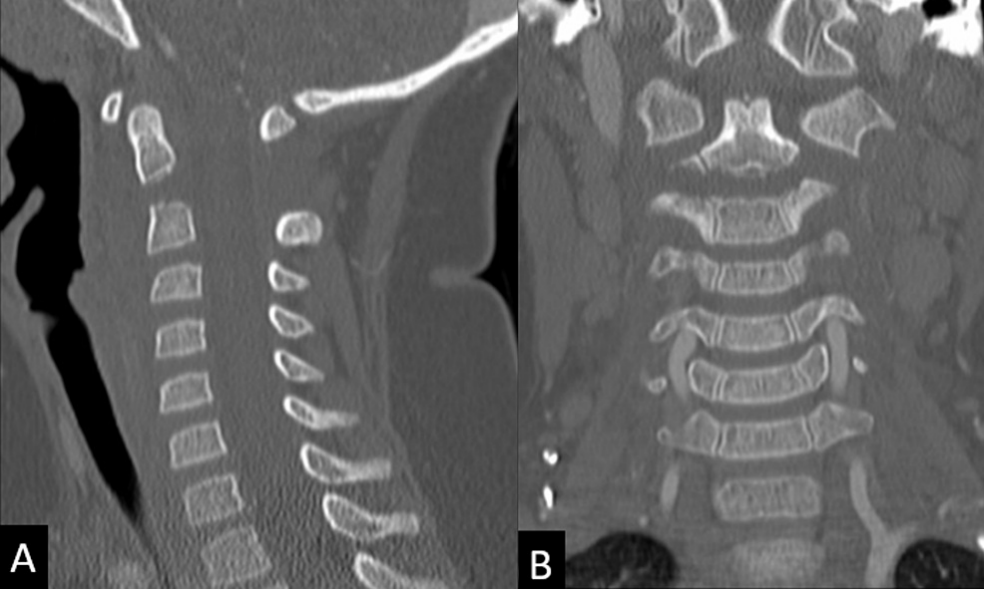
Sagittal (A) and coronal (B) CT cervical spine images demonstrating a fracture through the central odontoid synchondrosis with posterior angulation and 5mm of distraction.

A three-year-old female presented after a motor vehicle collision where she was a seat-belted rear passenger in a forward-facing booster seat. She complained of neck pain but had a normal neurological exam and no other identified injuries. The evaluation revealed a displaced and angulated odontoid synchondrosis fracture with associated ligamentous disruption (Figures 1A, 1B). External halo bracing was attempted with poor fracture reduction, in part due to the patient having an obese body habitus and baseline behavioral disorder preventing adequate immobilization.

**Figure 2:**
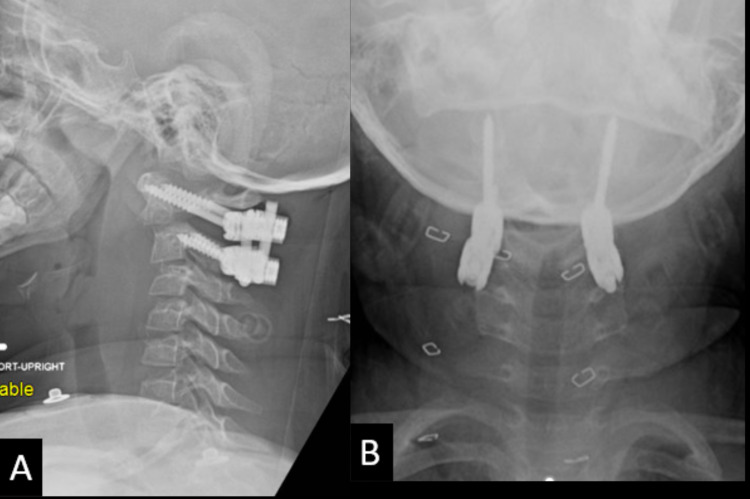
Lateral (A) and anterior-posterior (B) upright cervical spine plain films demonstrating placement of C1 lateral mass screws and C2 pars screws with improved fracture alignment and appropriate hardware placement.

Surgical reduction and stabilization were recommended. Posterior C1-2 instrumentation was placed with C1 lateral mass and C2 pars screws for rigid fixation (Figures 2A, 2B). Intraoperative neuromonitoring and fluoroscopy-guided reduction were carried out while stereotactic navigation was used for screw placement. We avoided joint capsule disruption or arthrodesis. Four months later, with imaging confirmation of bony fracture union, the instrumentation was removed without difficulty. She had no complications from either surgery.

**Figure 3:**
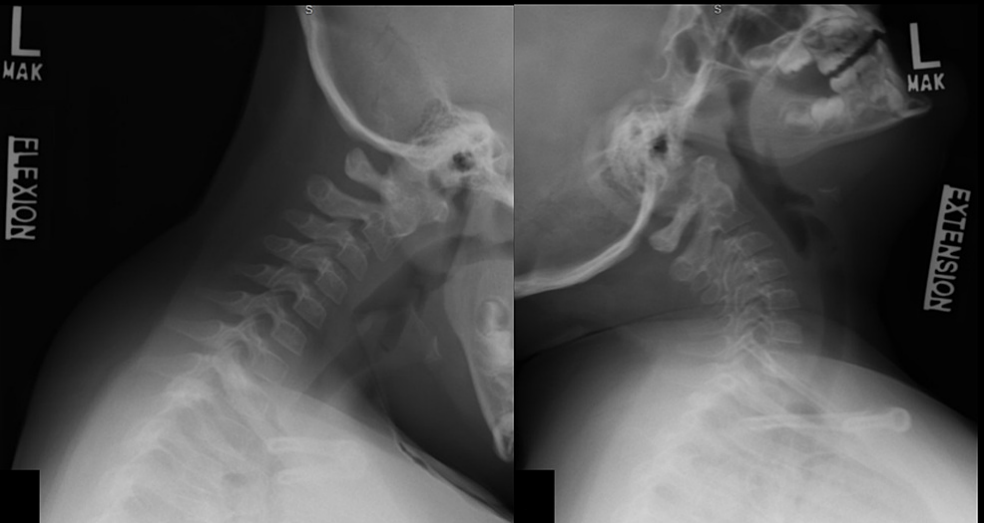
Lateral views of flexion-extension plain films of the cervical spine demonstrating no evidence of instability at the C1/2 junction after removal of hardware.

Postoperatively, she has done well with over one year of follow-up. She has no neck pain or neurological deficits. Her cervical range of motion (flexion/extension and atlantoaxial) is preserved on the exam, and dynamic imaging at 12 months postop demonstrated normal atlantoaxial motion with no evidence of instability (Figure 3).

## Discussion

Anatomic development of C2 consists of five primary and two secondary ossification centers; children are at greater risk for odontoid synchondrosis fractures due to increased head-to-body ratio, lack of supporting musculature, ligamentous laxity, and incomplete ossification of the synchondroses. Ossification at the craniocervical junction is incomplete until the later stages of childhood (> eight years old), thus rendering this region particularly vulnerable to fractures in high-energy trauma [8]. Although the overall incidence of spinal injuries is lower in young children relative to the adult population, management of pediatric cervical spine fractures deserves special consideration due to the greater associated morbidity and mortality [10].

The primary treatment for the majority of odontoid synchondrosis fractures is external immobilization, which is successful in over 90% of patients [11]. Surgery is generally indicated for fracture angulation of more than 30 degrees, evidence of upper spinal cord injury, displacement of the odontoid of 11% or more, or failure of conservative management [12]. In the reported literature, surgical treatment includes a permanent fusion of C1-2, with lifelong alterations to cervical biomechanics and range of motion.

Temporary instrumentation allows the benefits of surgical reduction and rigid stabilization without the long-term implications of fusion. With the demonstration of successful fracture management in this patient, this technique can be considered for appropriately selected children with odontoid synchondrosis fractures.

## Conclusions

Odontoid fractures are among the most common cervical spine injuries in children, though these fractures are often able to be managed in a closed fashion when adequate reduction is unable to be obtained operative management follows. Currently, the most commonly employed operative approach to fixing these fractures results in permanent bony fusion and loss of atlantoaxial ROM. In our case, we illustrate the utility of temporary internal bracing which may be successfully accomplished via temporary C1-2 instrumentation without arthrodesis, avoiding the lifelong implications of fusion.
